# The five continuum of care criteria that should accompany rectal artesunate interventions: lessons learned from an implementation study in Malawi

**DOI:** 10.1186/s12936-023-04514-5

**Published:** 2023-03-25

**Authors:** Monique S. Oliff, Pamela Muniina, Kenneth Babigumira, John Phuka, Hans Rietveld, John Sande, Humphreys Nsona, Maud M. Lugand

**Affiliations:** 1 WellSense Public Health, Kilifi, Kenya; 2 College of Medicine, University of Malawi, Blantyre, Malawi; 3 Medicines for Malaria Venture, MMV, Geneva, Switzerland; 4 National Malaria Control Programme, Ministry of Health, Lilongwe, Malawi; 5 Integrated Management of Childhood Illness (IMCI), Ministry of Health, Lilongwe, Malawi

**Keywords:** Severe malaria, Continuum of care, Pre-referral treatment, Rectal artesunate, Community health worker, Africa, Referral mechanisms

## Abstract

**Background:**

Rectal artesunate (RAS) is a World Health Organization (WHO) recommended intervention that can save lives of children 6 years and younger suffering from severe malaria and living in remote areas. Access to RAS and a referral system that ensures continuity of care remains a challenge in low resource countries, raising concerns around the value of this intervention. The objective of this study was to inform RAS programming, using practical tools to enhance severe malaria continuum of care when encountered at community level.

**Methods:**

A single country two-arm-controlled study was conducted in Malawi, where pre-referral interventions are provided by community health workers (CHWs). The study populations consisted of 9 and 14 village health clinics (VHCs) respectively, including all households with children 5 years and younger. CHWs in the intervention arm were trained using a field-tested toolkit and the community had access to information, education, and communication (IEC) mounted throughout the zone. The community in the control arm had access to routine care only. Both study arms were provided with a dedicated referral booklet for danger signs, as a standard of care.

**Results:**

The study identified five continuum of care criteria (5 CoC Framework) to reinforce RAS programming: (1) care transitions emerged as to be dependent on a strong cue to action and proximity to an operational VHC with a resident CHWs; (2) consistency of supplies assured the population of the VHC’s functionality for severe danger signs management; (3) comprehensiveness care ensured correct assessment and dosing; (4) connectivity of care between all tiers using the referral slip was feasible and perceived positively by caregivers and CHWs and (5) communication between providers from different points of care. Compliance was high throughout but optimized when administered by a sensitized CHW. Over 93% experienced a rapid improvement in the status of their child post RAS.

**Conclusion:**

RAS cannot operate within a vacuum. The impact of this lifesaving intervention can be easily lost, unless administered as part of a system-based approach. Taken together, the 5CC Framework, identified in this study, provides a structure for future RAS practice guidelines.

*Trial registration number and date of registration* PACTR201906720882512- June 20, 2019.

## Background

The risk of death of a child from severe malaria is greatest in the first 24 h after developing danger signs and requires parenteral treatment without delay. In malaria-endemic countries, the transit time between referral and arrival at a health facility where parenteral treatment can be administered can exceed 6 h [1]. To avoid deterioration or death, a child living remotely must access the closest primary-care touch point (village health post or clinic—VHC) and receive artesunate as a rectal capsule, as soon as danger signs of severe malaria have been identified by the community health worker (CHW) [1]. RAS comes in a softgel rectal capsule with a consistent thermostable shape. Capsules can go through repeated cycles of melting and solidifying which does not damage either the inert fill or the capsule shell. This is fundamentally different from waxy suppositories which do not have a consistent thermostable shape. The child must then be referred, without delay, for continued treatment at a higher-level facility within 24 h. The referral is only successful once the patient has been received, logged as a severely ill patient post RAS, and slotted into a severe malaria case-management trajectory, following strict guidelines: parenteral artesunate as an in-patient, to manage the severe malaria for at least 24 h or until the patient can tolerate oral therapy, treatment for other conditions contributing to the severely ill status, followed by a 3-day course of oral artemisinin-based combination therapy (ACT) [1, 2].

An effective referral system from the community village health clinic to and into the referral healthcare facility is the basis of the continuum of care (CoC) [3, 4] that ensures continuity of care for the sick child with severe malaria, until complete recovery [2]. Overcoming the barriers to successful referral and working towards full recovery can go a long way to safeguarding access to, and long-term efficacy [5] of this lifesaving intervention for the hardest-to-reach communities of sub-Saharan Africa.

The World Health Organization (WHO) recognizes that a well-functioning referral system is a pivotal component of primary healthcare, particularly in rural areas with poor communication facilities [6]. It describes an effective referral system as being comprised of referral flows: an initiating facility that follows a protocol of care, uses an outward referral form to link with a receiving facility where, upon receiving the client with a referral form, treats the patient and provides feedback to the initiating facility [7]. Health surveillance assistants (HSAs) are the first point of contact in Malawi and serve as a link between the community and the health facility [8]. With 12 weeks of basic training covering all the competencies to manage health issues at the community level [8], they serve as a low-cost measure for delivery of basic health support in hard-to-reach areas [9–11], serving populations of up to around 1,000 to 3,000 people with support from village health committee volunteers [8, 12]. They typically reside in their catchment area and provide promotional, preventive, or curative healthcare, among other tasks on integrated Community Case Management (iCCM) of childhood illnesses [8, 12]. In Malawi, the HSAs, like other non-voluntary CHWs across the region, are an essential cadre in driving forward efforts to achieve universal health coverage. As a result, their duties and responsibilities are constantly expanding [13] and they must rely heavily on referrals to the second and third tiers of the health system for complex cases. Finding ways to enhance the quality of their work and to strengthen and formalize their links to the other levels of care is essential in order to reinforce the CoC [11, 14]. iCCM relies on a strong referral system for severely sick children [15] at risk from complications or death [14, 16], however there is a paucity of literature focusing on factors that contribute to an effective referral originating from community health systems [17–19]. This includes specific ways to strengthen the referral system upon which hard-to-reach, low-resource settings rely, such as the ones in this study, when young children present with severe febrile illness.

A referral from a peripheral village clinic is complex and multifaceted. A referral can fail at any point along the continuum, due to any number of barriers arising from the patient/caregiver/community and the health system [20]. These include transportation problems (no access or cost); costs or care at the referral facility; family-related reasons (e.g., mother also ill, lack of a carer for the other children at home, social structures in which women’s movements are restricted) [16, 21, 22] and concerns about poor reception or poor quality of care at the referral facility (e.g., unskilled staff, unavailability of medicines, inappropriate response) [23–25]. Similarly, factors that relate to access to clinical information [26], health personnel skills in referral counselling of the caregiver (emphasizing urgency and that RAS is not a full treatment), perception of the purpose of the drug (ACT for uncomplicated malaria versus RAS for severe malaria dangers signs) and the state of the child (perceived urgency) can influence successful referral [27].

Studies in Malawi have revealed limited awareness of severe malaria symptoms at referral health facilities, as well as a perception of treatment guidelines being a suggestion rather than a requirement demanding strict adherence [28]. The lack of understanding of severe malaria among receiving health workers, specifically how to identify and manage a child post-RAS (who may no longer show danger signs) as per national guidelines including post-treatment follow up, can thwart any successful referral from the community post RAS [28]. If some health workers at higher level facilities are choosing not to follow the recommendation (guidelines) to admit patients presenting with danger signs or with a recent history of danger signs, then dedicated programmatic strategies that urgently redress health worker deficiencies in severe malaria management are essential [29–32].

Within a system-thinking framework, each group either intercepts or facilitates a successful referral and in turn either enables or disables the continuity of care. The need to strengthen the health system highlights the importance of these relationships and preempts the inherent unpredictability when these components interact [33]. Nevertheless, focusing on factors that are directly within the control of the health system such as health worker behaviour, has proven to be very powerful. While verbal referrals tend to diminish the strength of a referral, a written referral slip combined with a recommendation to the caretaker to immediately seek referral care and highlighting the consequences of not doing so, is a key factor in referral completion [20, 23].

A responsive and well-functioning referral system is a cornerstone of the CoC for severely ill vulnerable children [34] who are at higher risk of death [35]. The objective of this study was to inform future rectal artesunate programming, using practical tools to enhance the CoC, originating at the household and ending up back at the household, with a child free of acute illness. Considering the debate around the risks and benefits of rectal artesunate as lifesaving pre-referral interventions in remote settings, this paper shares practical measures that reduce delays before and after accessing the village health clinic, when dangers signs are present, as well as systems-based recommendations that ultimately enhance the CoC without which the RAS intervention is of no use.

## Methods

The study was conducted in Malawi, in the Salima and Ntchisi districts. Malawi is a high-burden malaria country with an incidence rate of 332 cases annually per every 1000 people and approximately 4.8 million episodes of malaria per year [36]. In Malawi, 82.6% of the population live in rural areas [37], and up to 35% of children aged 5 and under rely on the village health clinic (VHC) as their closest point of care [38]. The village health clinic is the first level of the Malawian 5-tier healthcare system and in closest reach of rural and hard-to-reach communities. It is manned by health surveillance assistants (HSAs), equivalent to a paid community health worker. The two districts where the study was implemented are both moderate-to-high transmission settings in the Central Region of Malawi, with the highest burden of severe malaria in children of this age group of interest at the time of the study. The study population represented the hardest-to-reach village health clinics (VHCs) with a resident community health worker and included households located more than five kilometres from a government referral health facility. This criteria of hard-to-reach area was guided by the WHO definition, alongside the WHO recommendation that the RAS has the most significant positive effect on children living furthest from care, specifically those living 6 h from the nearest health facility [39, 40]. The study was initiated towards the end of the malaria season 2018/2019 and continued for 12 months until the end of March 2020.

**Fig. 1:**
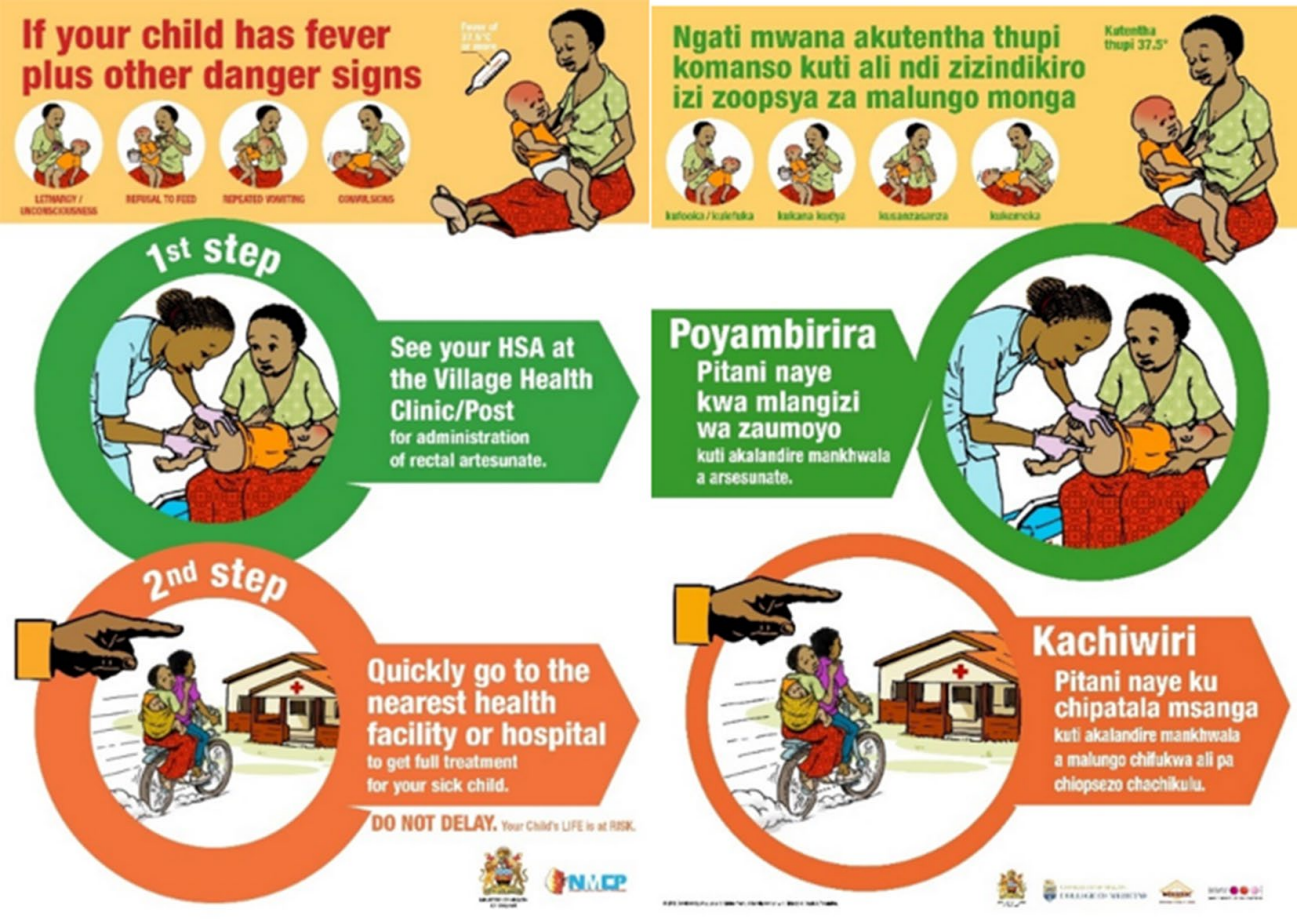
Examples of community sensitization posters (English and Chichewa)

**Fig. 2:**
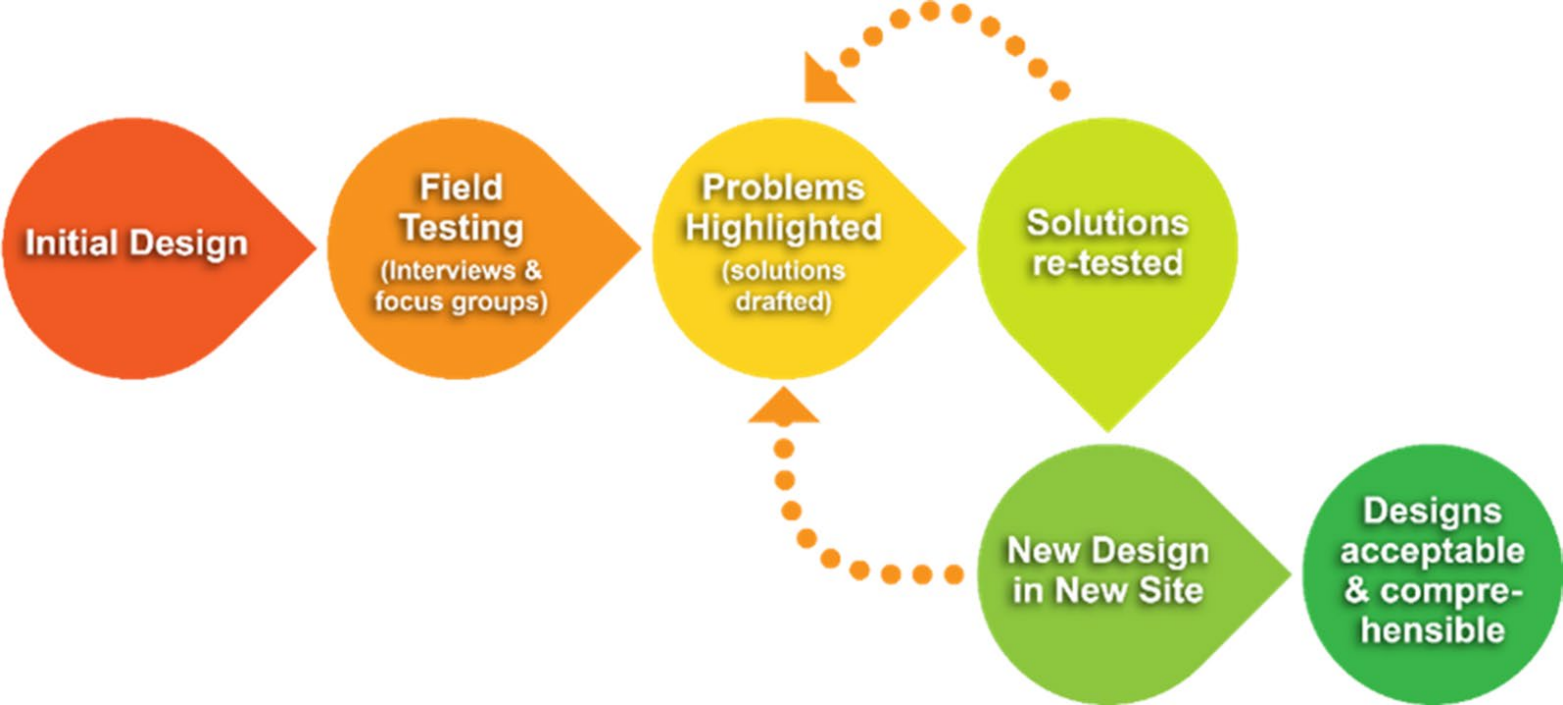
Participatory iterative qualitative design of the IEC toolkit

It was designed as a single country, two-arm study. The control arm consisted of 9 VHC zones, which managed severe malaria as per routine national guidelines, whilst the 14 VHC zones in the intervention arm were exposed to a RAS information, education, and communication (IEC) toolkit (Fig. 1), which formed part of the social behaviour change intervention. All HSAs working in the 14 hard-to-reach VHC zones were enrolled in a half-day refresher session, to familiarize them with the elements of the IEC toolkit: job aid, reference booklet, flip chart. These tools were to be used during clinical or educative encounter sessions with community members. In the control arm, neither the community, nor the HSAs in the 9 VHC zones received any additional RAS-related IEC. The tools were developed prior to the study and field tested in Senegal and Malawi, using a participatory iterative qualitative process (Fig. 2) [41].

The VHCs (via their HSAs) in both districts, procured RAS supplies through government channels (pulled from the health centre as need). If during supervision, the HSA was found not to be procuring the commodity from the referral centres, they were prompted to restock by the study team. In addition, both sites received a carbon copy referral booklet, developed for rectal artesunate referrals use only. The decision to include the referral booklet at both sites was made for two reasons: (1) to add an additional tool to track the patients’ trajectory along the continuum of care, and (2) to formalize the referral mechanism and enhance linkages between the community and health facility level, irrespective of the study arm. The integrated management of childhood illness (IMCI) referral slips were not in operation or out of stock when the study was launched and throughout the duration of the study (Fig. 4).

**Fig. 3:**
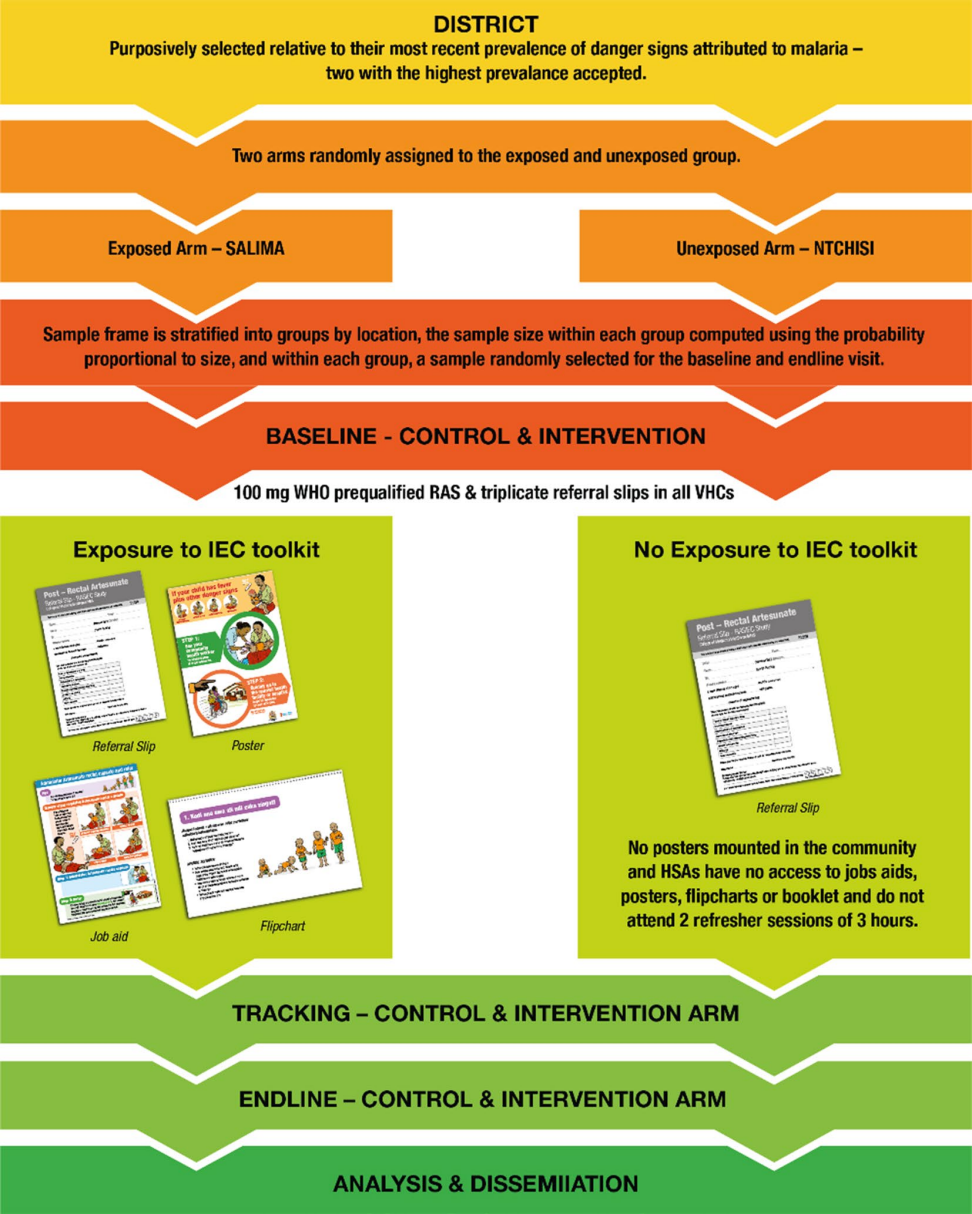
Study design

**Fig. 4:**
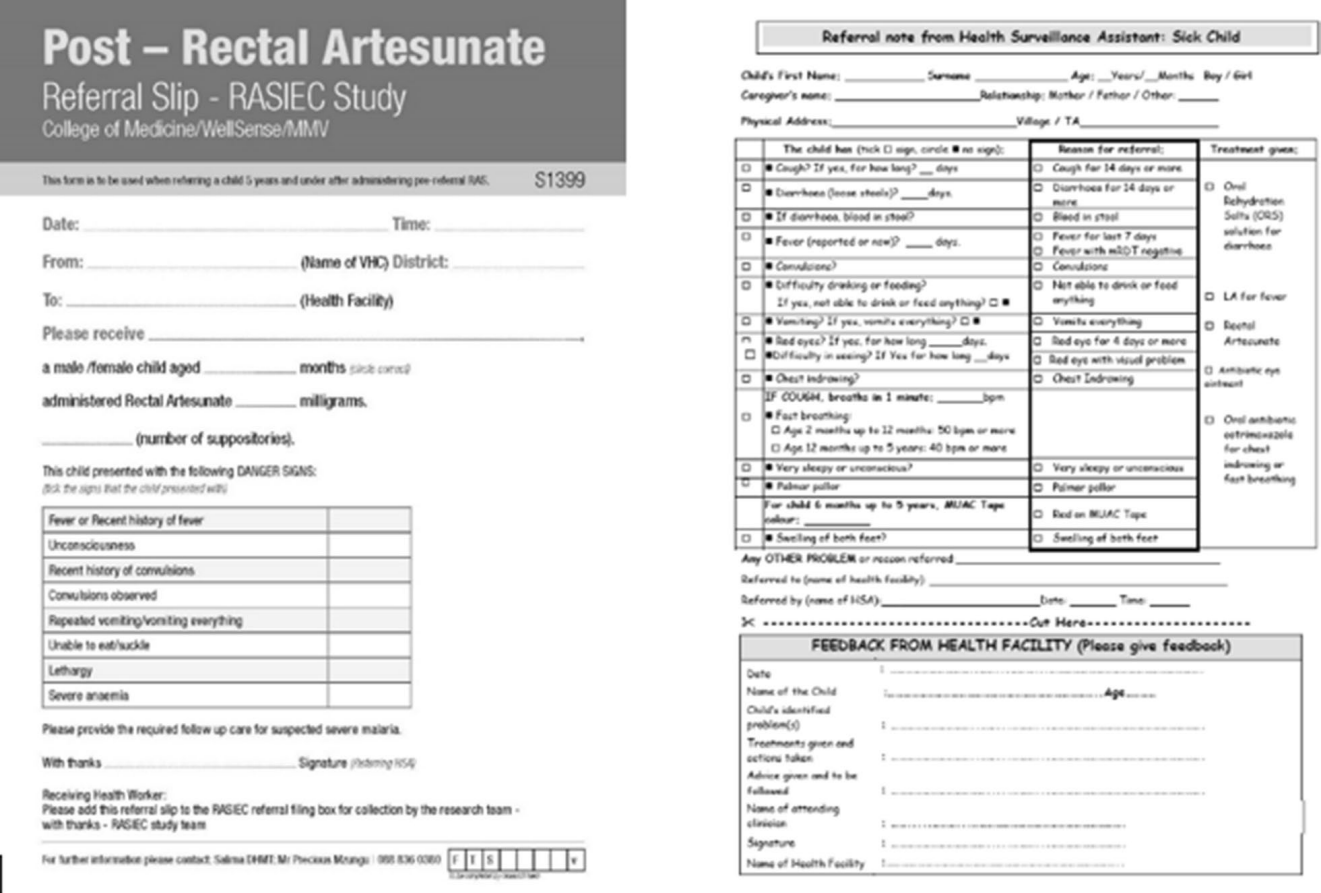
Referral slips (study) and Ministry of Health IMCI referral slip not in use during the study

Both study arms were investigated at baseline and then observed, to determine the attitudes, behaviour and practices of the caregiver and HSAs in response to an event of suspected severe malaria, either in the household or at the VHC respectively, with or without possible exposure (community), and confirmed exposure (HSA/VHC) to the toolkit and/or other components of the intervention (Fig. 3). All VHCs, in both study arms were exposed to the study referral slip (Fig. 4).

The sample size of households was calculated in relation to the incidence of danger signs/severe febrile illness reported at the health facilities, within the catchment areas of the study during 2018, and adjusted according to the likelihood of the caregiver to seek medical services. This provided a sample of the households that were expected to have an incident of severe malaria during the subsequent malaria season, relative to the approximate number of households within the catchment area of each VHC. The design effect (adjusting for VHC effect—with households clustered under the VHCs) was found to be 1. The same households were interviewed at baseline and at endline. Though replacement at baseline was acceptable, the response rate was included in the computation of the sample size to accommodate for refusal or unavailability of a household who was interviewed at baseline, at endline.

The households included in the baseline/endline met the following criteria:

- Household is within the catchment population of the selected hard-to-reach VHC.
- Household is home to at least one child 5 years and under.
- A primary caregiver is available and consents to be interviewed.

A sample consisting of 173 (exposed) households in Salima and 55 (unexposed) households in Ntchisi was established. The sample was selected randomly from the (study) arms divided into quarters, to ensure an equal representation of households across the catchment area. The sample of households in each quarter was proportionate to the number of total households in the quarter and the households were then selected at random, within that quarter.

All HSA activities in relation to every case of severe febrile illness (SFI) presenting at the VHC with regards a child 5 years and under, were recorded. This was followed up by a tracking visit with the caregiver no less than 7 days after the reported VHC visit. The primary caregiver was interviewed in relation to the most recent episode of febrile illness when the child was taken to the VHC. All 23 HSAs were contacted each day (via a message or a call) throughout the tracking phase of the study (9 months). During phone tracking, the HSA reported the actions s/he took during that encounter with the febrile child. These actions were recorded and then triangulated with the information recorded on the referral slip during the encounter and with the caregiver report, obtained during the follow-up interview at the homestead.

Originally the sample size for the tracking component was based on Health Management Information System data compiled from the previous year’s records, however within the first months of the study it became evident that this target was unreachable and could not be verified. Instead, the target number of predicted severe febrile illness/danger sign cases was redetermined and derived from the incidences of SFI reported at the referral centres within the study catchment areas, and the rate of increase of reported incidences at the referral centres in 2018–2019, using the monthly increments. The same rate of change (monthly increments) was applied, using the number of actual cases recorded between May and end August 2019, in order to predict how many cases could present for the study to track over the subsequent months. The study tracked the entire ‘population’ of caregivers seeking care for paediatric SFI and danger signs at the VHCs (all incidences of SFI/danger signs reporting to the VHC from June 2019 to February 2020) and recorded/reported by the HSA for the selected period. The study captured 57 (intervention arm) and 31 (control arm) SFI cases from the VHC records for parametric analysis.

### Recruitment

#### Health surveillance assistants (HSAs)

All HSAs from all the eligible village health clinics in the project areas in the two study arms were recruited for the study.

#### Severe febrile cases

All cases of severe febrile illness with danger signs that visited and that were reported to the enrolled VHCs were listed. The enrollment in the tracking phase took place once the case was reported by the HSA.

#### Caregivers for baseline/endline survey

A random sample of households (with at least one child 5 years or under) was compiled using each HSA household listing of their catchment area by village name, alongside the prevalence of SFI cases from the previous malaria season. The exact location of the household was known to health volunteers who were responsible for recording the number of children 5 years and younger in each household in their jurisdiction within the catchment area.

### Analysis

#### Baseline/endline phase

Descriptive analyses of predefined indicators were performed at baseline assessment, once during the tracking phase, and once at endline, 9 months after initiation of the intervention. A comparison between baseline and endline caregiver’s knowledge, attitudes, and practices, and the perception of community attitudes at endline were undertaken to create a variable that defined whether the level improved, deteriorated, or remained the same. The exposed and unexposed arms were compared by this variable to observe if there was evidence of a significant difference in the proportions, then adjusted for the potential covariates identified at baseline, undertaking a logistic regression.

#### Tracking phase

The actions taken by the HSA during the visit were extracted from details noted in the HSA self-report, gathered during phone tracking, and triangulated with (1) the referral tracking slips and (2) the caregiver report during the follow-up home visit.

The household tracking assessments primarily investigated the level of recognition and appropriate response to danger signs, RAS knowledge, and compliance with the HSA referral and the nature of the care received at the referral facility, after referral, was complete. These indicators were collected for the tracked households in both study arms and their comparison was adjusted depending on the characteristics of the caregiver, the child’s age, and perceived severity of the child’s illness. With the variables in the comparison grouped into categorical variables, logistic regression was undertaken. The activities of the HSAs during the caregivers’ visit to the VHC were assessed and the proportion of caregivers that received care in line with the guidelines in the exposed arm was compared, relative to those in the unexposed arm.

## Results

It is well established that rectal artesunate is a lifesaving intervention. This study identified five continuum of care criteria (5CC Framework) that should feature as part of any RAS implementation guideline: care transitions for the patient; consistency of supplies at delivery sites; comprehensiveness of care received by the patient at the VHC and at referral health centres; connectivity of care between providers; and communication between all points of care. The data to support this recommendation is presented below:

### Care transitions for the paediatric patient and caregiver

#### Cue to action

The transition from homestead to the village health clinic with a sick child often requires a stimulus to trigger a decision-making process, referred to as a cue to action. Project posters provided pictorial information on severe malaria danger signs and indicated where care could be sought. At endline, 80% of the respondents in the exposed arm reported seeing at least one of the 200 study posters. They were displayed in various locations which included meeting places, on prominent trees on well-used paths and on leaders’ houses (Fig. 1). Most respondents reported that they were unable to read but responded positively to the pictorially based posters. 92.4% of respondents who had seen the poster understood it, determined by their capacity to recall the key learning content of the poster during interview. These posters became a valued cue to action in the exposed arm over the course of the intervention period. In the control arm, the most significant reported cues to action were knowledge acquired through word of mouth that severe malaria medicines were now available in the village clinic (which was not the case prior to the study) and through knowledge of a death of a child in the village from malaria/danger signs (Table 1).

**Table 1:** Household reported cue to action in response to severe malaria

Cue to action	Exposed n = 173		Unexposed n = 55		Comparison p value	Exposed		Unexposed		Comparison p value
	**#**	**%**	**#**	**%**		**#**	**%**	**#**	**%**	
Posters and pictures mounted in the village/my community	97	56.1	50	90.9	< 0.001	158	95.8	18	32.7	< 0.001
Knowledge of availability of medicine for danger signs at the VHC	51	30.0	9	16.4	< 0.001	96	58.2	53	96.4	< 0.001
The death of a child from malaria/danger signs in the village	86	50.0	8	14.6	< 0.001	109	66.1	51	92.7	< 0.001

### Care transitions

#### Closest primary-care point of care

A successful care transition from homestead to the village health clinic is founded on the desire for residents in the catchment area to access care at the nearest primary-care point. At the study sites, 97% of all respondents knew about the VHC, with 85% having visited their health centre in the past year, primarily due to a child’s illness. The VHC was the first and preferred point of care for severe childhood febrile illness, primarily due to ease/proximity (80% reported they could reach the VHC within 1 h by foot), adequacy/speed of access and because tertiary health facilities (and even some health centres) have policies restricting self-referrals. Two-thirds of caregivers were seen swiftly, spending only half an hour at the VHC, including waiting time.

### Consistency of supplies at delivery sites

#### VHC readiness—functionality and conduciveness to delivery of RAS

Knowledge that the village health centre is functional and that the necessary commodities are available, motivates people to transition from homestead to the nearest primary point of care. This assessment was made over the course of 276 monitoring visits throughout the duration of the study. The data revealed significant infrastructure challenges at the village health clinics, including inconsistent access or lack of clean water for handwashing, inconsistent supplies of gloves, absence of lighting for night-time care, as well as unreliable access to dry secure storage for commodities. In addition, malaria-related clinical supervision was very low to absent throughout the 12-month study; 93% of the monitoring visits revealed no on-site supervision had taken place during the preceding month. Prior to the launch of the study, RAS was not routinely available in the study VHCs. Knowledge that RAS was now available at the VHC was reported as a cue to seek care among community members. Although the study did not supply the commodity, the study ensured that RAS was always in the VHCs, in both study arms. When, during routine monitoring, it was noted that RAS supplies were diminishing, the HSAs/CHWs were prompted to pull the commodity from the health centre as per district procedures. RAS was administered to 100% of the cases with danger signs presenting at the VHCs. Knowledge that RAS was regularly available (consistency of supplies) proved to be a powerful cue to seek care for a child with danger signs and resulted in the artesunate being administered to every case.

### Comprehensiveness of care at the VHC

#### Care delivery

Another criterion of continuum of care highlighted in this study, relates to the nature of the care provided and whether it was considered ‘comprehensive care’ and appropriate in relation to the presenting symptoms. The toolkit and HSA sensitization training emphasized the need for comprehensive care, defined as—*child examined/assessed, RAS administered (correct dose, correct method), referral slip & instructions given—*and encouraged HSAs to have the job aid to hand when managing a child with signs of severe malaria. There was strong evidence that children with danger signs (Table 2), the majority of whom had multiple danger signs at one time who were managed by sensitized HSAs with access to the job aid, had a significantly higher chance of receiving comprehensive care. The social behaviour change intervention had a positive impact on correct response and administration of RAS among exposed CHWs/HSAs. This group of CHWs showed higher levels of knowledge and offered a superior quality of care, in comparison to the unexposed CHWs. This kind of simple change requires consistent supervision in order to be sustained.

**Table 2:** Severe malaria danger signs that presented at the village health clinic

Danger signs	#	%
Unable to feed/drink	41	46.1
Severe vomiting	32	36.0
Altered consciousness	16	18.0
Convulsions or history of convulsions	42	47.2
Extreme weakness	11	12.4
Severe anaemia	9	10.1

Total respondents n = 89—one or more responses reported

When comparing the association between the age of the child and the service provided, both the mean and median show that the likelihood of receiving correct care from the HSA is greater among sick children who are younger. In addition, there is evidence that the odds of correct services being offered increases with a decrease in the age of the sick child—odds ratio 0.95) CI: 0.92–0.98).

#### Dosing of rectal artesunate

**Fig. 5:**
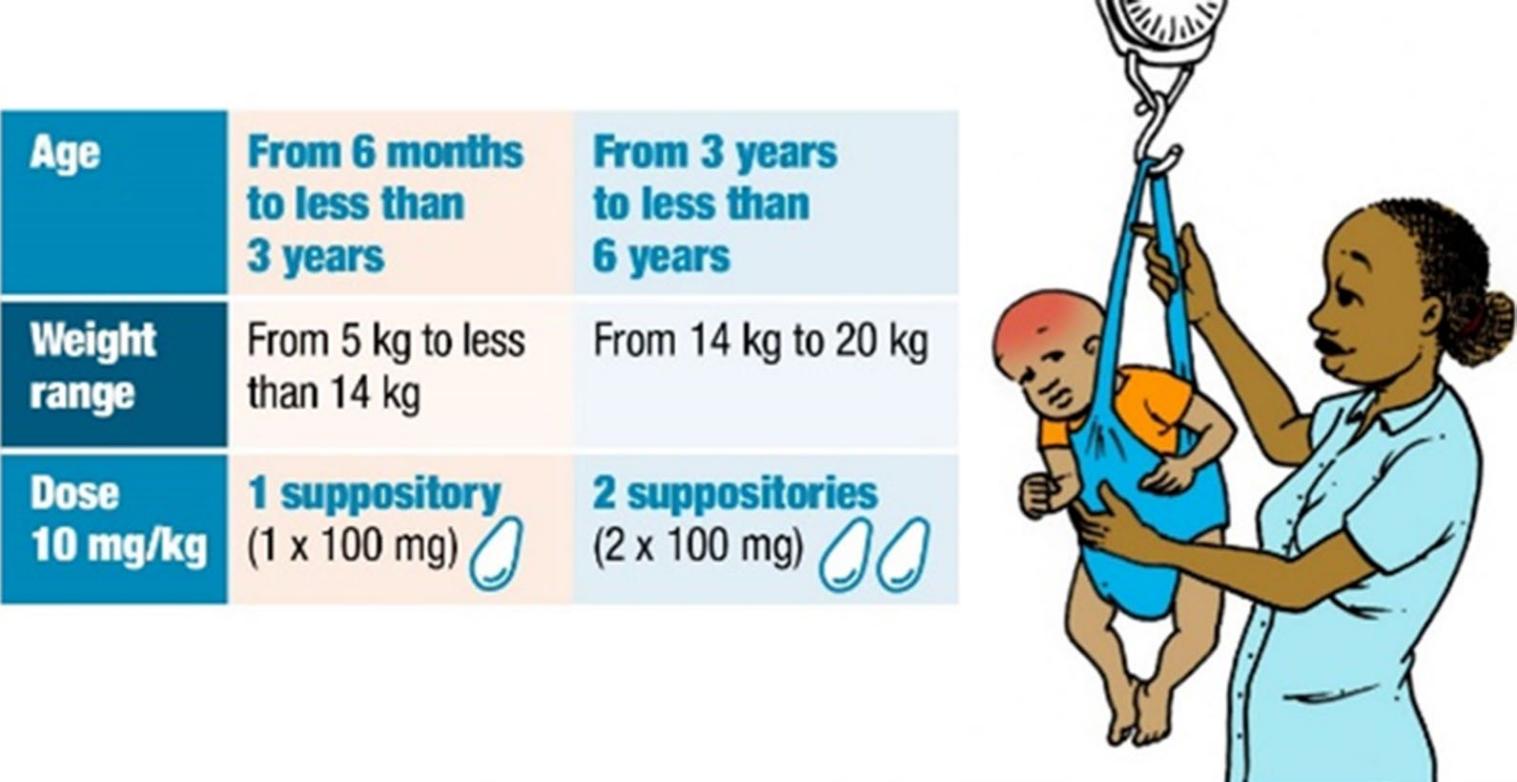
Example of dosing schedule from job aid (available in Chichewa for study purpose)

A key component of comprehensive care is the assurance that the patient will receive the correct dose (Fig. 5). Most children were administered the correct dose based on their age. In the exposed arm, 84.5% of the cases were administered the correct dosage, while in the unexposed arm, 71.9% were given the correct dosage. The tendency was to under-dose and administer one capsule instead of two. As weighing scales were not readily available, the children were not weighed and dosing was, therefore, based solely on estimated age. The HSAs in the intervention sites had access to a dosing table but in 16% of cases the HSAs administered the incorrect dose, solely based on the age of the child. Whether the HSA adjusted the dose downward because they determined that the child’s weight was lower, they underestimated the age of the child, or whether this was a clinical error, was not determined.

### Connectivity of care

#### Role of referral slips

Continuity of care requires a dependable and consistent way of enhancing connectivity of care between providers and the different levels within the health system. The referral slip performed this role, irrespective of the availability of the RASIEC toolkit. All referrals were counter-verified during routine monitoring by checking the referral slip duplicate retained in the referral slip book. The referral was then tracked to the health facility (where the majority were retrieved) and others were retrieved at the homestead during the follow-up interview. The dedicated/condition-specific referral slips proved to be an intervention in both study arms that was well received by HSAs and caregivers alike. Although not officially part of the toolkit, it was perceived as such and caregivers in both arms (84.4% in unexposed; 94.4% in exposed) reported that the referral slip had a ‘positive’ impact on the response they received at the referral health facility. The HSAs reported that the referral slip book was a key tool, as it legitimized, formalized and authenticated their referrals and, as a result, the caregivers took the stipulation to seek advanced care more seriously than with a verbal referral.

#### Caregiver compliance with referral

The success of *connected care* depends on caregiver compliance with the health worker’s recommendation for action which, in this case, is referral for post-RAS treatment. Compliance with referral was extremely high and essentially equal in both arms—over 96% in both. Additional sensitization of the HSA/CHW did not affect whether the caregiver adhered to referral or not. Caregivers reported rapid change in the state of the child soon after RAS was administered (93.99% in unexposed; 92.9% in exposed). Despite this improvement, caregivers did not delay complying with the referral.

#### Time taken to reach care after referral

The transit time between referral and arrival at a health facility, influences how connectivity of care is evaluated*.* In the intervention site, it was rapid, with 89% reaching the referral centre within the stipulated 24-h window, and 74% reaching the referral facility in less than 5 h. Caregivers whose children were treated by sensitized HSAs/CHWs—trained to ‘assess’–‘treat’–‘refer’ –, displayed a greater response to time pressure but the difference was not significant. The referral slip was a very effective tool in encouraging caregivers to travel with urgency.

### Comprehensiveness of care at the referral centre

#### Care received at the referral centre

Once a child arrives at the referral centre, the assumption is that he will receive the correct post-RAS care. The study did not evaluate the care provided at the referral centre, but data was gathered based on the caregiver’s recall. Admission for a minimum of 24 h is expected after RAS administration for signs of severe malaria, to allow for observation during the administration of parenteral artesunate. Of key interest, in relation to WHO guidelines for post-RAS management of severe malaria, was whether the child was admitted (versus treated as an outpatient), and whether the child received parenteral treatment: intravenous (IV) drip/injection (Table 3).

**Table 3:** Care (parenteral IV or injection) & admission at the referral facility (caregiver recall)

Number of danger signs	IV/Drip or Injection	Admitted	Total	Percent
One (1) danger sign	No	No	3	3.4
		Yes	0	0.0
	Yes	No	4	4.5
		Yes	4	4.5
Two (2) danger signs	No	No	22	24.7
		Yes	1	1.1
	Yes	No	7	7.9
		Yes	8	9.0
Three (3) danger signs	No	No	21	23.6
		Yes	1	1.1
	Yes	No	2	2.2
		Yes	7	7.9
Four (4) danger signs	No	No	9	10.1
		Yes	0	0.0
	Yes	No	0	0.0
		Yes	0	0.0
Total			89	100.0

Of the 86 caregivers (primarily mothers) who responded to this question, 21 (24.5%) reported that their child was admitted as an inpatient, and that they spent nights at the hospital (something a caregiver is very likely to recall). Of all the tracked cases who received RAS and complied with referral, only 30% received parenteral care at the referral facility, note that some were administered parenteral treatment on an outpatient basis and did not receive the three doses of injectable artesunate followed by artemisinin-based combination therapy as required by national guidelines. This lack of alignment with severe malaria treatment guidelines to admit the patient to administer post-RAS treatment, revealed the need to further explore this dimension of the continuity of care.

## Discussion

Considering the debate around the risks and benefits of rectal artesunate as a lifesaving pre-referral intervention for remote communities, this study set out to find ways to enhance the continuum of care. The study asked whether a bespoke IEC package (as part of a social and behaviour change process), targeting hard-to-reach community members and first-line health workers in their local language, improved danger sign recognition and response. This paper sheds light on practical (toolkits and referral slips) as well as systems-based measures that can not only reduce delays before and after accessing the village health clinic, when dangers signs are present, but have the potential to reinforce the referral and communication continuum and capitalize on the benefits of RAS.

The limitations of the study were fourfold: the use of the referral form as a standard of care requirement across both the control and intervention sites and not as an intervention in its own right; the focus of the evaluation on only one tier of the health system—village health clinic (VHC)—at the exclusion of the referral health centre; the introduction of the malaria vaccine trial in the control district during the course of the study, and the reliance on caregiver recall to describe the care trajectory of the sick child.

Referral forms were considered a standard of practice and in theory are included in the HSA practice manual but were not in use or available in the study sites throughout the 12 months of the study. Since a written referral is considered a standard of practice, dedicated danger sign referral forms were supplied by the study in both the intervention and control sites as an adjunct to care and as a method to track the care trajectory. Therefore, in the original study design, the referral slip was not introduced as an intervention in its own right, which restricted the scope of the analysis as there was no study arm operating without the referral slip. With regard to the second limitation, the study was neither designed nor funded to evaluate post-RAS care at the referral facility. The assumption was made that where the injectable artesunate commodity was available, treatment guidelines for a child with a history of danger signs would be adhered to. The third limitation relates to the introduction of the Malaria Vaccine Trial (MVT), which was launched in the district housing the control arm of this study, after the baseline was completed. This may have influenced the heightened levels of knowledge and health seeking in response to danger signs in the control arm, resulting in some bias in the unexposed areas where the MVT was underway—possibly diluting the apparent effects in the intervention arm. The final limitation is with regard to the reliance on caregiver recall, to provide some insight into the care trajectory post RAS and the care received upon arrival at the referral centre. Ideally the study would have had the resources to expand its scope to the referral centre and triangulate the caregiver response with direct observations and/or record review. Although maternal recall for recent and acute events has been validated as a methodology [42, 43], particularly in relation to unambiguous questions, such as those asked ‘was your child admitted—did you spend nights at the hospital?’ and ‘did your child receive injections or drips as part of the care provided at the referral centre?’ (both memorable events for the mother of a young child). The study recognizes that the responses of the caregiver provide limited insight into the scope of the care provided and reasons why the referral centre clinicians chose not to admit and treat the majority of the children referred post RAS.

Despite more than 80% coverage and associated poster visualization and information recall, the posters did not result in community members in the intervention arm seeking care at the VHC for danger signs more than their counterparts in the control arm with no access to the posters. It emerged that in general, caregivers in both study arms did not delay care once their child presented with danger signs, the delay in seeking care, occurred before the child developed danger signs. This emphasized the need for IEC materials that alert caregivers to the more subtle signs and symptoms of malaria in order to avoid a life-threatening situation. The findings that community members understood and could recall the content from the tailor-made posters, was encouraging and relevant to future interventions.

The study had hypothesized that the presence of an IEC toolkit among HSAs operating in a setting with minimal on-the-job supervision, would enhance knowledge and practice among HSAs, specifically in relation to the management of malaria danger signs. HSAs who had been exposed to the IEC toolkit did show higher levels of knowledge and their practice was positively influenced; overall, they offered a higher and more comprehensive quality of care compared to that of the HSAs in the control arm.

The introduction of regular supplies of RAS to all the VHCs in the study area resulted in consistent availability of a commodity to which access had previously been unreliable. This in itself had a significant influence on residents seeking healthcare. Information circulating by word of mouth (observed in both the study arms), makes community members aware of an available and effective service, indirectly influenced awareness, knowledge and even practice in both study arms.

The introduction of a carbon copy referral slip booklet alongside the RAS commodity (in the absence of the Ministry of Health’s form) proved to be a very useful tool for both the caregiver and the HSAs, influencing the care, compliance, and reception at the referral health centre. The combination of the two components—the commodity and the referral slips—played an important role in enhancing the continuum of care and closing the gap from VHC to referral facility.

The study demonstrated that when the caregiver is counselled about the importance of immediate referral and given a dedicated slip, there is a very good likelihood that the child will reach the referral centre/hospital within 24 h. A functional referral system is one of the cornerstones of the continuum of care [34] for sick children. At the outset, caregivers are not likely to understand the hierarchical structures of the health system and the need for referral. Patients who successfully transfer to higher levels of care are carrying key information in the form of a referral slip from an HSA who has identified danger signs and administered RAS. The referral slip should be reviewed by a qualified clinician at the referral centre, who should guide the treatment plan as per national guidelines.

However, the study revealed that despite 96% of referral completion upon arrival at the referral health centre, only 20% of caregivers reported that their child was admitted and 30% reported that their child received some form of parenteral treatment—two events that a caregiver could easily recall 7 days after the event [42]. Caregiver recall suggests that the care these children received was not in line with severe malaria treatment guidelines. Were some of these children not receiving the correct severe malaria treatment because they no longer presented with acute danger signs due to the success of RAS? If the child ‘appeared to be well,’ was the clinician assessing the patient and taking adequate note of the child’s history and reading the referral note?

The study findings suggest that investments made upstream, at the VHC, to save life and guard against monotherapy through effective danger sign assessment, pre-referral treatment and effective referral (as demonstrated by the completion rate), can be quickly lost downstream, if the patient who has travelled long distances at significant expense, and is expecting to be admitted, arrives at the referral centre outpatient department, and is not admitted. These impressions were based on caregiver recall and were not triangulated, however, they suggest that the risk of RAS-associated monotherapy does not lie solely in the periphery, but also with the care the children receive post RAS at the higher tiers of the health system.

Clinical recognition of severe malaria illness before and after RAS is different. This study’s intervention focused on quality IEC that outlined key danger signs of severe malaria and actions to be taken by the CHW before and after RAS. The study did not include training at health facility/referral level which could have had a significant impact on the level of care received. At baseline and throughout the study, none of the VHC sites in the study were routinely using referral forms in practice, and there was no referral register at the sending or receiving end of the continuum. Referred patients from the VHC are typically referred verbally and not in writing, so are not recorded and do not, therefore, appear in reports submitted officially to the district level [21].

The national malaria control and integrated management of childhood illness departments already recognize the importance of referral forms. They have developed a simple formalized referral slip dedicated to sick children (Fig. 4); however, it is not always available and, therefore, cannot be relied upon to facilitate the continuum of care. Just like drug commodities, it must be in stock and available at all times. A referral mechanism that supports a two-way referral along the clinical pathways will go a very long way to enhance the continuum of care. Encouraging countries to ensure that referral forms are available, are completed, received correctly, and followed up, could contribute to protecting this lifesaving intervention. If availability of referral forms cannot be guaranteed, then they should be included as an insert with the drug, as a low-cost mechanism to formalize referral and optimize child health outcomes.

CHWs are uniquely positioned as a link between the community and the health system as they are encouraged to live within the communities with which they have day-to-day interactions. CHWs are the agents for change working in these communities, and efforts must be made to invest in their resources and motivation: their home and clinic infrastructure, their access to information and tools that enable them to support equitable access to lifesaving, pre-referral interventions, and to successful referrals to the level of care available to less remote communities living near to referral centres [10, 11]. The success of any referral will in part depend on the community health worker—whether they are available during an emergency, their level of confidence, their access to information, guidance and supervision and the extent to which they feel that they are part of the health system. They need to feel valued and that what they do affects what happens downstream from their VHC and that their efforts are worthy of feedback.

Success will also depend on the economic implications for a family to follow through with a referral and to manage the unexpected costs of transport, food and accommodation and having to leave other children at home. Caregivers need to understand that malaria advances quickly in a child and that accessing care earlier in the illness will save money and inconvenience—different kinds of IEC to those used in this study are needed to communicate these messages. Most importantly, the success of the intervention will depend on how the child is received at the referral centre and if the child is triaged and managed properly, with a complete history and a referral slip that is used by the receiving clinician that highlights the need for correct management post rectal artesunate, based on the status of the child prior to receiving RAS.

### Recommendations

The field-tested tailor-made job aids influenced quality of care and should be accompanied by regular clinical supervision by Ministry of Health staff. The referral slips, however, emerged as the intervention with the most impact. This study revealed that the referral slip rendered the referral non optional and was valued by both caregivers and the CHWs/HSAs. As such, it suggests that rectal artesunate should only be administered by CHWs who have access to referral slips. The dangers of non-referral should be emphasized on the packaging and directed at the CHW administering the drug.

This simple low-cost referral slip addressed one of the key concerns with RAS at the outset of the study: poor compliance with referral post rectal artesunate and the risk of monotherapy. The preliminary findings that the majority of children tracked in this study were not admitted and/or administered any parenteral treatment post RAS as reported by caregivers, raised the need to study referral centre treatment practices and the choices clinicians make on how to manage a referred child. Referral centre health workers need to be very familiar with the interventions taking place upstream and need to be guided to anticipate the referral slip as well as use the information on the referral slip to guide their treatment plan.

A formal, low-cost referral mechanism that emphasizes connectivity of care must be considered as part of a basic standard of care for febrile children being referred to higher levels of care. Introducing referral slips to enhance the continuum of care served as a powerful linkage tool for provider and patient and reinforces five continuum of care criteria (5CC Framework) that emerged from this study. However, connectivity of care requires that caregivers recognize signs and transition without delay; that the commodity is available—serving as a cue to action; that the health surveillance assistant is well versed in comprehensive assessment, RAS dosing and care; that the referral slip connects the points of care through the patient and finally that the feedback loop reinforces communication between all points of care, ensuring that the benefits of any one intervention are not lost either upstream nor downstream.

## Conclusion

Malawi continues to use RAS as a pre-referral intervention in hard-to-reach communities for children presenting with fever and danger signs of severe febrile illness. Between January and June 2022, 5, 234 children with fever and one or more danger signs, were referred to a health facility for further management. Of these, 4558 cases (87%) received a pre-referral dose of RAS.

The objective of this study was to inform future rectal artesunate programming, introducing social and behaviour change tools while looking to enhance the continuum of care. In light of the debate around the risks and benefits of rectal artesunate as lifesaving pre-referral interventions in remote settings, this paper highlights that the RAS intervention cannot operate within a vacuum. The impact of this lifesaving intervention for the dangerously ill child living in a hard-to-reach community can be easily lost, unless it is administered as part of a system-based approach. Taken together, the 5CC Continuum of Care Framework, identified in this study, provides a structure for future RAS practice guidelines.

## Data Availability

The datasets supporting the conclusions of this article are publicly available from https://rasiec.mmv.org/.

## Abbreviations

ACTs: Artemisinin combination therapy
CHWs: Community health workers
CoC: Continuum of care
ICCM: Integrated community case management
IEC: Information education and communication
HSA: Health surveillance assistant
RAS: Rectal artesunate
VHCs: Village health clinics
WHO: World Health Organization
